# Critical Melting–Freezing Pretreatment Enhances Enzymatic Hydrolysis for Porous Starch Preparation: Role of Partial Structural Weakening and Surface Modification

**DOI:** 10.3390/foods14172984

**Published:** 2025-08-26

**Authors:** Chen Zhang, Chu-Yun Wu, Shi-Qi Qian, Yu-Yan Zhang, Ya-Li Liu, Xin-Yu Li, Shi-Yi Wang, Jian-Ya Qian

**Affiliations:** 1 School of Food Science and Engineering, Yangzhou University, Huayang Xilu 196, Yangzhou 225127, China; mx120241324@stu.yzu.edu.cn (C.-Y.W.); 233601219@stu.yzu.edu.cn (S.-Q.Q.); mz120232141@stu.yzu.edu.cn (Y.-Y.Z.); mx120241341@stu.yzu.edu.cn (Y.-L.L.); mx120231326@stu.yzu.edu.cn (X.-Y.L.); 18752198045@163.com (S.-Y.W.); 2 Postdoctoral Mobile Station of Agriculture, College of Agriculture, Yangzhou University, Wenhui Donglu 48, Yangzhou 225009, China

**Keywords:** porous starch, melting, enzymatic hydrolysis, encapsulation, structure

## Abstract

In this study, critical melting followed by freeze–thaw (CMFT) pretreatment was employed as an effective strategy to partially weaken and modify the surface structure of starch, enhancing enzymatic hydrolysis (EH) for porous starch preparation. Compared with EH alone, the CMFT + EH treatment synergistically facilitated porous structure formation while preserving structural integrity. Partial structural weakening and surface modifications induced by CMFT promoted enzyme diffusion into amorphous starch domains, enabling efficient hydrolysis and pore development without excessive granule degradation. CMFT + EH treatment reduced enzyme requirements and hydrolysis time by 33% compared to single enzymatic hydrolysis while markedly increasing water and oil absorption capacities. Porous starch prepared by CMFT + EH exhibited enhanced ordering of double-helical structures, with RC% increasing from 25.48% (native) and 24.74% (enzymatic hydrolysis alone) to approximately 28%. Furthermore, CMFT + EH significantly improved curcumin encapsulation efficiency from 40% (native) to ~88% and increased curcumin stability under various storage conditions. This study provided an effective strategy to enhance enzymatic hydrolysis efficiency for porous starch preparation with reduced enzyme addition and hydrolysis time.

## 1. Introduction

Porous starch, one of the modified starches characterized by high specific surface area with pores/channels on surfaces and/or within granule interiors, is widely utilized as an absorbent, encapsulation agent, and protective carrier in food and biopharmaceutical applications [1,2,3]. Among various preparation methods (physical, chemical, and biological), enzymatic hydrolysis is increasingly favored as an eco-friendly biological approach and widely used for porous starch preparation [4].

Although enzymatically hydrolyzed porous starch typically exhibits superior pore structures compared to physicochemical methods, it also has two critical application limitations that need to be addressed. (1) Non-uniform pore formation: Random attachment of enzyme molecules to starch granules commonly causes uneven hydrolysis, resulting in low porosity, heterogeneous pore size distribution, and localized structural collapse [2,5]. While increasing enzyme concentrations or optimizing hydrolysis conditions may promote pore development, these approaches concomitantly induce excessive granule degradation, thereby limiting porous starch applications. (2) Loss of granular integrity: Research over recent decades has primarily focused on promoting pore development through various treatments [2,6]. However, maintaining granular integrity remains equally critical, as the encapsulation efficiency and controlled release performance of bioactive compounds fundamentally depend on intact granular structure during processing and storage. Although pore formation inevitably disrupts structural integrity, achieving a balance between enzymatic efficiency and granular integrity remains important for porous starch preparation.

To address these limitations, researchers increasingly combined enzymatic hydrolysis with some pretreatments (e.g., freeze–thawing, sonication, HMT, and ANN treatment) to enhance porous starch production. These pretreatments partially weaken internal starch structures, thereby facilitating enzyme accessibility and pore formation [7,8]. Among physical methods, freeze–thaw (FT) treatment has been reported as an effective strategy for porous starch preparation, utilizing ice crystal-induced mechanical forces to generate surface grooves or pores [2,5,9,10]. During FT treatment, mechanical forces generated by ice crystal expansion/contraction locally compress starch granules, weakening starch inner structure and creating surface grooves/pores or expanding micropore channels [1,2,3,5,9,10]. However, FT efficacy highly depends on starch water content, as it determines ice crystal size, distribution, and the generation of mechanical forces. Recently, critical melting at the onset temperature of starch before FT (CMFT) has been reported as an effective way to enhance FT effects by promoting starch swelling and facilitating water penetration into the granule interior [5,10]. Critical melting spatially confines water penetration to pre-swollen amorphous domains, partially disrupting hydrogen bonds in amorphous lamellae while preserving crystalline integrity, thereby creating “soft zones” for ice crystal growth and promoting pore development without loss of granular stability. Subsequent FT treatment thus generates ice crystals that preferentially expand within these hydrated amorphous regions, avoiding excessive disruption of crystalline lamellae and balancing pore development with granular stability. Based on these mechanisms, it can be hypothesized that FT and CMFT pretreatments could increase enzyme attachment sites by weakening internal structures of starch granules and creating surface grooves and pores, thereby enhancing enzyme accessibility to facilitate porous structure formation while maintaining granular integrity.

In this study, normal maize starch was selected as the primary research material owing to its unique structural attributes, which inherently contain surface-penetrating micropores (50–300 nm diameter) as compared with other non-porous starches (e.g., potato or wheat starch). These pre-existing channels facilitate enhanced water infiltration during hydrothermal treatment, promoting ice crystal formation that generates directional mechanical forces to expand pore architecture. For porous starch preparation, FT and CMFT pretreatments were applied prior to enzymatic hydrolysis to partially weaken the compact inner structure of starch and create surface grooves or pores for enzyme binding, potentially reducing enzyme requirements. The morphology, functionalities, and structural characteristics of the prepared porous starches were investigated. The synergistic mechanism of physical-enzymatic treatment for porous starch preparation was elucidated. Furthermore, the encapsulation efficiency and stability of curcumin within the prepared porous starch were systematically investigated. Curcumin, a natural polyphenol derived from turmeric (*Curcuma longa*), exhibits potent antioxidant, anti-inflammatory, and chemopreventive properties, but its instability and poor aqueous solubility significantly limit its application. By combining physical treatments and enzymatic strategies, this work will provide an effective strategy for producing porous starch with defined pore structures while preserving granular integrity.

## 2. Materials and Methods

### 2.1. Materials and Reagents

Normal maize starch (containing 22% amylose) was purchased from Zhucheng Xingmao Corn Developing Co., Ltd. (Zhucheng, Shandong, China). α-Amylase (BAN 480 L, activity 2792 U/g) was provided by Novozymes (Beijing, China). Pepsin (3000 U/g, catalog# S10030-25 g) and pancreatin (4000 U/g, catalog# S10031-25 g) were obtained from Yuanye Biological Technology Co., Ltd. (Shanghai, China). Curcumin (catalog# C805205-5 g) was purchased from Macklin Biochemical Technology Co., Ltd. (Shanghai, China). Soybean oil was purchased from a local market (Yangzhou, China). All reagents and chemicals were of analytical grade.

### 2.2. Preparation of Porous Starch

#### 2.2.1. Physical Pretreatments

Pretreatments prior to enzymatic hydrolysis included the freeze–thaw (FT) treatment and critical melting with FT (CMFT).

(1). Freeze–Thaw (FT): Starch was initially dispersed in deionized water at a ratio of 2:3 (*w*/*w*). The suspension was then frozen at −20 °C (slow freezing rate, ~0.08 °C/min) or −80 °C (rapid freezing rate, ~2.0 °C/min) for 4 h, respectively, followed by being thawed at 25 °C for 1 h in a water bath.

(2). Critical Melting and Freeze–Thaw (CMFT): Starch was dispersed in deionized water at a ratio of 2:3 (*w*/*w*) and then heated in a water bath at the onset melting temperature of starch (named as critical melting, CM, To: 63 °C) for 1 h with magnetic stirring. The *T*o used in this study was premeasured using a differential scanning calorimeter (DSC 8500, PerkinElmer, Waltham, MA, USA). Subsequently, the dispersion was freeze–thawed as described above.

All pretreated starches were dried overnight at 40 °C in a convection oven, ground into powder, and sieved through a 75 μm mesh for subsequent enzymatic hydrolysis.

#### 2.2.2. FT/CMFT Pretreatment Combined with Enzymatic Hydrolysis

NMS and pretreated starches were individually dispersed in 80 mL of sodium hydrogen phosphate–citrate buffer at a concentration of 25% (*w*/*v*), and α-amylase was added to each dispersion. The optimal temperature for enzymatic hydrolysis is 50 °C. Then, the sample was enzymatically hydrolyzed at the premeasured optimal hydrolysis conditions (Supplementary Figures S1–S3). Optimal hydrolysis conditions for native starch are 120 U/g of α-amylase for 12 h at pH 6.0, while the optimal hydrolysis conditions for FT- and CMFT-pretreated starches are 80 U/g of α-amylase for 8 h at pH 6.0. After enzymatic hydrolysis, a hydrochloric acid solution (1 mol/L, 2 mL) was used to stop the enzyme reactions. Subsequently, the samples were subjected to centrifugation three times at 1610× *g* for 10 min, dried at 40 °C for 8 h, and then ground and sieved (75 μm).

For better comparison of the effects of combined treatments (FT + EH and CMFT + EH) and the single enzymatic hydrolysis (EH), two control groups were established. Native starch hydrolyzed at 120 U/g for 12 h at pH 6.0 was set as “Control A” (EH^120^), while native starch hydrolyzed at 80 U/g for 8 h at pH 6.0 was set as “Control B” (EH^80^). The experimental design and corresponding nomenclature are summarized in Table 1.

### 2.3. Pore-Forming Properties of Porous Starch

#### 2.3.1. Surface Microstructure

The internal structural characteristics of native and porous starches were observed by an Emission Scanning Electron Microscope (GeminiSEM 300, Carl Zeiss, Jena, Germany) at an accelerating voltage of 5.0 kV after being coated with gold under a vacuum (BAL-TEC SCD 500, Balzers, Liechtenstein) before observation. The micrographs were taken at 4000×.

#### 2.3.2. Internal Structural Features

The internal structural features of native and porous starches were observed using confocal laser scanning microscopy (CLSM, 880NLO, Carl Zeiss Microimaging GmbH, Jena, Germany) according to the method reported by Monroy, Rivero, and García (2018) [11]. The samples were stained with the fluorophore APTS (8-amino-1,3,6-pyrenetrisulfonic acid) and scanned.

#### 2.3.3. Specific Surface Area, Pore Size Distribution, and Total Pore Volume

The specific surface area, pore size distribution, and total pore volume of both native and porous starches were determined through low-temperature liquid nitrogen adsorption at −196 °C using the Brunauer–Emmett–Teller (BET, Autosorb-IQ3, Quantanchrome Instruments, Boynton Beach, FL, USA), following the procedures described by Guo et al. (2020) [12] with minor modifications. The samples were degassed at 120 °C for 5 h under a vacuum. Then, the sample was placed into the specific surface area and porosity analyzer after being cooled to room temperature. High-purity nitrogen was used as the adsorption medium. The specific surface area was calculated using the BET (Brunauer–Emmett–Teller) equation. The monolayer value (a_m_) was calculated within the range of p/p_0_ = 0.06–0.20 using a linear polynomial form of the BET equation [13]. Additionally, pore size and volume were calculated using the BJH (Barret–Joyner–Halenda) method [14].

### 2.4. Functional Properties of Porous Starch

#### 2.4.1. Water (WA) and Oil (OA) Absorption

Starch (1.0 g) was mixed with 10.0 mL of deionized water or soybean oil and vortexed for 20 min. Then, the mixture was centrifuged at 1610× *g* for 30 min. The supernatant was removed, and the precipitate was weighed [15]. The water (WA%) and oil (OA%) absorption of starch were calculated according to Equation (1).

(1) $$\mathrm{WA}\%\;\mathrm{or}\;\mathrm{OA}\%=\left(\frac{W_{1}-W_{2}}{W_{2}}\right)\times 100\%$$

where W_2_ (g) is the initial weight of the sample before water or oil absorption and W_1_ (g) is the final weight of the sample after water or oil absorption.

#### 2.4.2. Swelling Power (SP), Water Solubility Index (WSI), and Water-Holding Capacity (WHC)

Native and porous starches were dispersed in deionized water at a concentration of 10 mg/g (dry basis) and subsequently heated at temperatures of 50, 70, and 90 °C for 30 min in a water bath. The samples were then centrifuged at 1610× *g* for 30 min, and the precipitate was weighed. The precipitate was weighed and dried at 105 °C to constant weight. The WSI (%), SP (g/g), and WHC (g/g) were calculated according to Equations (2)–(4), respectively.

(2) $$\mathrm{WSI}\;(\%)=\frac{W_{1}}{W}\times 100$$

(3) $$\mathrm{SP}\;(g/g)=\frac{W_{2}\times 100}{W(100-WSI)}$$

(4) $$\mathrm{WHC}\;\left(g/g\right)=\frac{W_{2}-W_{1}}{W_{1}}$$

where W (g) is the initial weight of the powder sample, W_1_ (g) is the weight of the dried supernatant, and W_2_ (g) is the weight of the precipitate after centrifugation.

#### 2.4.3. Differential Scanning Calorimetry (DSC)

The thermal properties of native and porous starches were determined using a differential scanning calorimeter (DSC 8500, PerkinElmer, Waltham, MA, USA). The powder sample (5 mg) was mixed with deionized water (10 μL) and then sealed in an aluminum pan. After being equilibrated overnight, the samples were scanned from 25 °C to 100 °C at a heating rate of 5 °C/min. The melting temperatures of onset (To), peak (Tp), conclusion (Tc), and enthalpy (ΔH) were determined from the thermograms.

### 2.5. Structural Characterization of Porous Starch

#### 2.5.1. X-Ray Diffractometry (XRD)

The crystalline pattern and relative crystallinity (RC%) of native and porous starches were characterized using an X-ray diffractometer (D8 Advance, Bruker AXS, Karlsruhe, Baden-Württemberg, Germany). Samples were loaded into a sample holder and then analyzed at a voltage of 40 kV and an electric current of 30 mA. Diffraction data was collected from 3 to 40° at a scanning rate of 4°/min. Relative crystallinity (RC%) was calculated using Jade software (Version 6.0, Materials Data, Inc., Livermore, CA, USA) according to Equation (5).

(5) $$\mathrm{RC}\;\left(\%\right)=\left(\frac{\mathrm{IC}}{IC+IA}\right)\times 100\%$$

where IC is the cumulative intensity of crystalline regions and IA is the cumulative intensity of the amorphous region.

#### 2.5.2. Small-Angle X-Ray Scattering (SAXS)

The lamellar structure of native and porous starches was investigated using a small-angle X-ray scattering system (NanoSTAR, Bruker AXS, Karlsruhe, Germany). The SAXS measurements were conducted at 50 mA and 50 kV with Cu Kα radiation (λ = 0.154 nm) as the X-ray source. The original spectral data was obtained with DIFFRAC plus Nano Fit software, and the data of the q value was collected from 0.2 to 1.4 nm^−1^. According to the Woolf–Bragg equation, the thickness of the starch semi-crystalline lamellar structure was calculated [16]. The peak area (Ap) was calculated using the software Origin 7.5, and the exponent α was derived from the slope of double-log ln*I*-ln*q* in the q range of 0.16–0.27 nm^−1^.

#### 2.5.3. Fourier Transform Infrared Spectroscopy (FTIR)

The short-range ordered structure of starch was analyzed using an ATR-FTIR spectrometer (Cary 610/670, Varian, Walnut Creek, CA, USA). The sample was mixed with KBr and pressed into a pallet. Spectra were recorded over the wavenumber range of 400–4000 cm^−1^ with a resolution of 4 cm^−1^ and averaged over 64 scans. The spectra were analyzed using OMNIC professional software (Thermo Nicolet Corp., Madison, WI, USA, version 8.3) following baseline correction and background subtraction. The absorbance intensity at R_1047/1022_ cm^−1^ was calculated from the deconvoluted spectra.

### 2.6. Porous Starch–Curcumin Complexes

#### 2.6.1. Preparation of Porous Starch–Curcumin Complexes

A curcumin solution was prepared by dissolving 10 mg curcumin in 50 mL of 70% anhydrous ethanol under dark conditions. Then, porous starch (1.00 g, dry basis) was suspended in the prepared curcumin solution (10 mL) and continuously stirred at 25 °C for 2 h to adsorption. The mixture was centrifuged at 2780× *g* for 5 min and filtered. The suspension was collected into a petri dish and dried in the dark at 40 °C for 24 h. The dried sample was ground into powder and stored in a brown glass bottle at 4 °C for further analyses.

#### 2.6.2. Encapsulation Efficiency (EE) and Encapsulation Capacity (EC) of Curcumin

EE and EC were determined by dispersing 50 mg of porous starch powder in 10 mL of curcumin solution (followed by method 2.6.1), followed by centrifugation at 2780× *g* for 10 min. The supernatant was analyzed for residual curcumin content using a UV/VIS spectrometer (Lambda 35 PerkinElmer, Waltham, MA, USA) at 425 nm. The EE (%) and EC (mg/g) were calculated according to Equations (6) and (7).

(6) $$\mathrm{EE}\;\left(\%\right)=\left(\frac{M1}{M}\right)\times 100\%$$

(7) $$\mathrm{EC}\;\left(\mathrm{mg}/g\right)=\left(\frac{M1}{M0}\right)$$

where M is the mass of starch, M1 is the mass of curcumin per unit mass of embedding material, and M0 is the mass of curcumin in the embedding material per unit mass obtained from the test.

#### 2.6.3. Stability of Encapsulated Curcumin in Porous Starch Under Different Storage and Temperatures

The thermal stability of porous starch–curcumin complexes was subjected to 40, 60, 80, and 120 °C. Meanwhile, storage stability of porous starch–curcumin complexes was assessed by storing samples under dark conditions for 5, 10, 15, and 20 d. Then, complex powders were dissolved in 70% ethanol (final volume was 100 mL) and centrifuged. The supernatant was diluted appropriately, and the absorbance retention (OD%) was calculated using Equation (8).

(8) $$\mathrm{OD}\;\left(\%\right)=\left(1-\frac{A0-A1}{A0}\right)\times 100\%$$

where A0 is the initial absorbance and A1 is the absorbance after storage.

### 2.7. Statistical Analysis

All technological treatments were performed in triplicate to ensure the reproducibility of the preparation process. All experiments were performed at least 3 times. The data obtained were subjected to variance analysis using Duncan’s multiple range test performed with SPSS 25 (SPSS Institute Inc., Cary, NC, USA). Origin 8.05 (Stat-Ease Inc., Minneapolis, MN, USA) was used to plot charts.

## 3. Results and Discussion

### 3.1. Surface and Internal Structure Features of Porous Starch Granules

**Figure 1:**
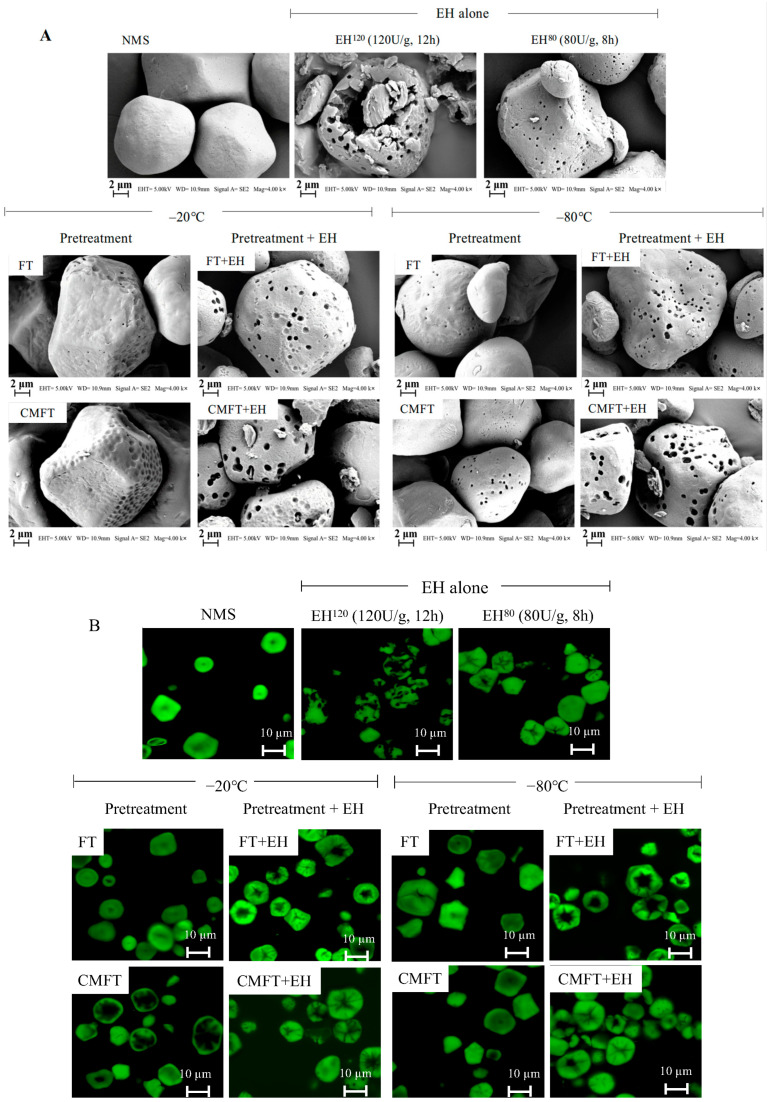

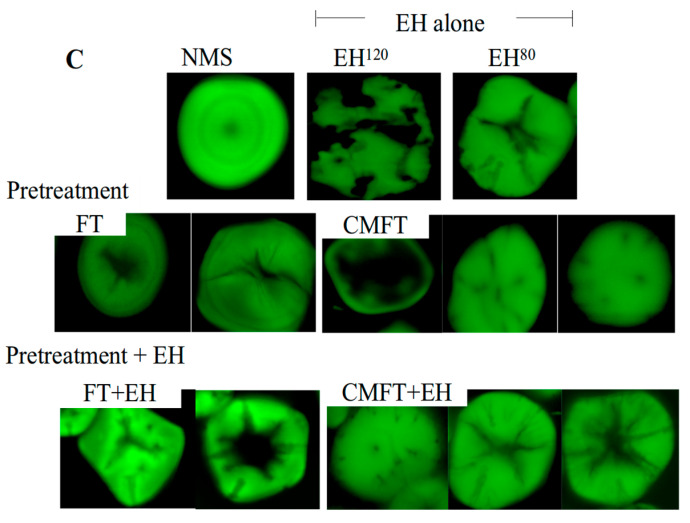
Surface microstructure (**A**) and internal structure features (**B**,**C**) of porous starch granules. (**C**) provides a representative magnified view from (**B**), providing clearer observation of internal structure features in porous starch. NMS indicates native maize starch. FT (freeze–thaw) and CMFT (critical melting with FT) indicate physical pretreatments before enzymatic hydrolysis. EH^120^ (control A: 120 U/g, 12 h, pH 6.0) and EH^80^ (control B: 80 U/g, 8 h, pH 6.0) indicate enzymatic hydrolysis alone. FT + EH and CMFT + EH indicate combined treatments. The freezing temperature includes −20 °C (slow freezing) and −80 °C (rapid freezing).

The surface and internal structural characteristics of porous starch granules are shown in Figure 1. Native starch granules (NMS) exhibited polygonal morphology with smooth surfaces and naturally enclosed internal structure [17]. Both FT and CMFT pretreatments modified surface structures and partially weakened internal structures of starch, generating surface grooves/pores (Figure 1A) and internal channels/cavities (Figure 1B,C). As shown in Figure 1A, native maize starch granules contained intrinsic microchannels that facilitated water diffusion into amorphous regions. Consequently, during FT treatment, ice crystals formed by water molecules generated mechanical forces that locally compressed starch granules, creating surface grooves and pores [10,17,18]. While ice crystals formed within granules (primarily in amorphous regions), they produced outward expansion forces that expanded channels or disrupted internal structures. CMFT further promoted porous structure formation by partially gelatinizing starch granules at its onset melting temperature. Critical melting treatment partially disrupted hydrogen bonds in amorphous lamellae, creating plasticized regions (“soft zones”) that improved water penetration and facilitated ice crystal growth during subsequent FT, promoting extensive pore and channel formation [18,19,20]. As shown in Figure 1, different freezing temperatures produced different porous structures in starch granules [3,9]. In detail, slow freezing (−20 °C) allowed for larger ice crystal growth, generating greater mechanical forces that produced wide surface grooves and internal cavities by partially damaging starch inner structures. Rapid freezing (−80 °C) formed small ice crystals within granule microchannels, producing transient mechanical swelling forces that expanded channels and formed deeper pores, particularly in CMFT-prepared samples.

Enzymatic hydrolysis alone (EH^120^ and EH^80^) further developed porous structures. EH^120^ (120 U/g, 12 h) created large pores but caused partial structural collapse and fragmentation due to random hydrolysis. Whereas EH^80^ (80 U/g, 8 h) preserved granular integrity through reduced enzyme addition and hydrolysis time, the mild hydrolysis conditions limited porous structure formation. Both granules’ excessive destruction (EH^120^) and insufficient porosity (EH^80^) may compromise the encapsulation efficiency of bioactive molecules within porous starch matrices.

Compared to enzymatic hydrolysis alone, porous starch prepared by FT + EH, particularly CMFT + EH (at both −20 °C and −80 °C), exhibited denser and deeper pores while maintaining granular integrity. As shown in Figure 1A, pre-formed surface grooves/holes and partially weakened internal structures by FT/CMFT provided additional enzyme attachment sites, thereby facilitating porous structure formation. Furthermore, these changes can also avoid enzyme molecules randomly attacking the granule surface or disrupting crystalline cores. As compared with EH^120^ (120 U/g, 12 h), both FT + EH and CMFT + EH (80 U/g, 8 h) significantly reduced enzyme addition and hydrolysis time. Figure 1 observations indicated that FT/CMFT pretreatments provided additional attachment sites and structural templates for enzymatic hydrolysis by partial weakening of structures and surface modification, thereby promoting porous formation without excessive granule degradation.

### 3.2. Starch Porosity

Table 2 summarizes the porosity characteristics of porous starch granules. The specific surface area, pore volume, and average pore diameter of NMS were 1.01 m^2^/g, 1.60 × 10^−3^ cm^3^/g, and 2.99 nm, respectively. Both FT and CMFT pretreatments significantly increased starch porosity. Freezing at −80 °C exhibited more significant increases in porosity than at −20 °C due to the differences in ice crystal formation dynamics, especially for CMFT. Enzymatic hydrolysis alone (EH^120^ and EH^80^) further increased starch porosity. In detail, EH^120^ increased these values to 17.00 m^2^/g, 15.97 × 10^−3^ cm^3^/g, and 3.44 nm, whereas EH^80^ increased them to 6.63 m^2^/g, 7.34 × 10^−3^ cm^3^/g, and 3.23 nm, respectively.

Combined treatments of FT + EH, particularly CMFT + EH, substantially increased starch porosity. For example, CMFT + EH increased specific surface areas and pore volumes by approximately 20–26 times compared with NMS and by 1–4 times compared with enzymatic hydrolysis alone (EH^120^ and EH^80^). These findings were consistent with SEM and CLSM observations, which revealed distinct grooves and pores on starch granules. The grooves and pores formed by FT and CMFT pretreatment provided specific sites for enzyme attachment, which minimized granule degradation and facilitated focused hydrolysis within amorphous domains, thereby improving the pore characteristics (e.g., increased pore volumes and specific surface area) of starch.

### 3.3. Water (WA%) and Oil (OA%)

Table 3 presents the water absorption (WA%) and oil absorption (OA%) of the starch samples. The WA% and OA% of NMS were 84.99% and 75.55%, respectively. Both FT and CMFT pretreatments significantly increased WA% to approximately 127% and OA% to approximately 76% (except for OA% in FT at −20 °C). This enhancement was primarily attributed to surface modifications of starch granules, specifically the formation of grooves and pores, which promoted the penetration of water and oil molecules. Furthermore, partial disruption of intermolecular hydrogen bonds in amorphous regions by FT/CMFT pretreatments weakened internal granular structures, thereby significantly enhancing starch absorption capacities. Freezing at both −20 °C and −80 °C increased WA% but slightly decreased OA%. This reduction in OA% was primarily caused by the limited pore dimensions (Figure 1) and partial granule disruption by ice crystals, which collectively inhibit penetration of large molecular weight oils into the granular matrix. Furthermore, FT induces syneresis in starch granules during water–ice phase transition, which causes starch chain reorganization and granular compression, thus further inhibiting oil molecule penetration.

Enzymatically hydrolyzed porous starch (EH^120^ and EH^80^) displayed substantially higher WA% and OA% than both NMS and pretreated starches. For example, EH^120^ increased WA% and OA% from 84.99% and 75.55% (NMS) to 150.25% and 120.40%, while EH^80^ increased to 121.05% and 94.38%, respectively. Compared with EH alone, combined treatment further increased WA% and OA% to approximately 150% and 120% (FT + EH) and to approximately 166% and 132% (CMFT + EH). This enhancement primarily stemmed from improved porous structure formation (Figure 1), with the preserved structural integrity of starch granules after combined treatments further facilitating water and oil absorption.

Previous studies reported that several physical-enzymatic strategies, such as microwave [8], hydrothermal [21], and extrusion [22], combined with enzymatic hydrolysis increased WA% and OA% within ranges of 140–160% and 60–150%. However, these combined treatments typically required more than 12 h for hydrolysis. In contrast, the present study showed that FT + EH and CMFT + EH increased WA% and OA% to approximately 170% and 130% within a significantly shorter hydrolysis time of only 8 h. Therefore, the combined treatments of FT + EH and CMFT + EH effectively promoted porous structure formation while preserving granular integrity, thereby providing a more efficient strategy for porous starch preparation with reduced enzyme addition and hydrolysis time.

### 3.4. Water Solubility Index (WSI), Swelling Power (SP), and Water-Holding Capacity (WHC)

**Figure 2:**
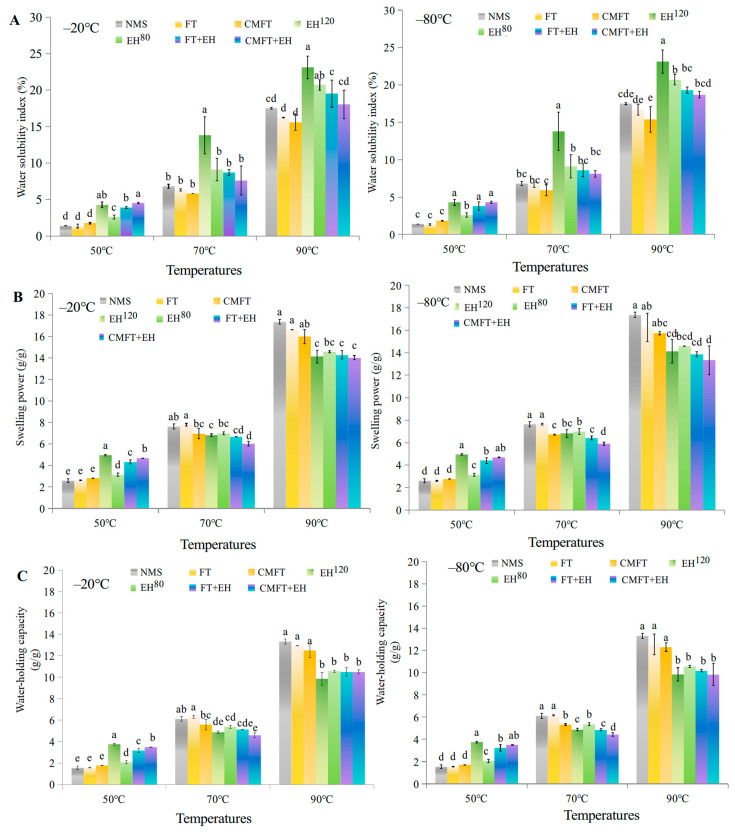
Water solubility index (**A**), swelling power (**B**), and water-holding capacity (**C**) of porous starch. Different lowercase superscripts (a–e) within bars indicate significant differences among treatments. NMS indicates native maize starch. FT (freeze–thaw) and CMFT (critical melting with FT) indicate physical pretreatments before enzymatic hydrolysis. EH^120^ (control A: 120 U/g, 12 h, pH 6.0) and EH^80^ (control B: 80 U/g, 8 h, pH 6.0) indicate enzymatic hydrolysis alone. FT + EH and CMFT + EH indicate combined treatments. The freezing temperature includes −20 °C (slow freezing) and −80 °C (rapid freezing).

The WSI, SP, and WHC of native and porous starches are presented in Figure 2. At 50 °C, both FT and CMFT pretreatments increased WSI, SP, and WHC compared to NMS due to pore formation. EH^120^ and EH^80^ further increased these parameters, with the highest increases observed after CMFT + EH and FT + EH treatments. Pre-formed pores and channels from FT/CMFT facilitated porous structure formation during the subsequent enzymatic hydrolysis, thereby enhancing water absorption [23]. These changes became more evident when starch was frozen at −20 °C than at −80 °C.

At 70 °C and 90 °C, WSI increased more rapidly in enzymatically hydrolyzed samples (EH^120^ and EH^80^) than in NMS and FT-/CMFT-treated counterparts. In general, porous starch increased WSI, SP, and WHC at higher temperatures because enzymatic hydrolysis weakened granular structural integrity and disrupted the water-binding layer of starch, increasing starch thermal sensitivity and structural degradation. However, porous starch prepared by both FT + EH and CMFT + EH exhibited lower WSI than that of EH^120^ and EH^80^. This reduction primarily resulted from molecular rearrangement in crystalline domains: critical melting induced starch swelling and released soluble starch from the amorphous regions, promoting starch inter-chain associations and granular syneresis, thereby inhibiting water absorption and swelling. Additionally, α-amylase cleavage of α-(1,4)-glycosidic bonds released low-swelling dextrins, further limiting thermal swelling.

Overall, porous starch prepared by FT + EH and CMFT + EH exhibited enhanced swelling and water absorption at 50 °C due to abundant porous structures. Meanwhile, as heating temperature increased to 70 °C and 90 °C, porous starch prepared by FT + EH and CMFT + EH exhibited increased thermal stability in swelling and solubility, suggesting a more compact internal structure.

### 3.5. Thermal Properties

Thermal properties of native and porous starches are summarized in Table 4. Compared to NMS, FT pretreatment increased both transition temperatures and ΔH, whereas CMFT increased transition temperatures but decreased ΔH, consistent with partial starch melting during critical melting. Enzymatic hydrolysis alone (EH^120^ and EH^80^) slightly increased transition temperatures while decreasing ΔH, indicating partial crystalline structure loss during hydrolysis.

Porous starch prepared via enzymatic hydrolysis typically exhibits reduced thermal stability due to pore formation and structural destruction [24]. In this study, the decreased ΔH in EH^120^ and EH^80^ also supported this general finding, indicating lower structural stability of porous starch. Compared with EH^120^ and EH^80^, FT + EH increased To, Tp, Tc, and ΔH, while CMFT + EH further increased these values. Partial structural weakening of both amorphous and crystalline structures during FT + EH/CMFT + EH released starch chains that easily reassociated into a denser and thermally stable network, thus requiring higher melting temperatures and energy [1,2,6]. Overall, the significantly enhanced transition temperatures and ΔH indicated that FT + EH, particularly CMFT + EH, enhanced the thermal stability of porous starch, suggesting their potential as heat-resistant adsorbents.

### 3.6. Crystalline Structure of Porous Starch

**Figure 3:**
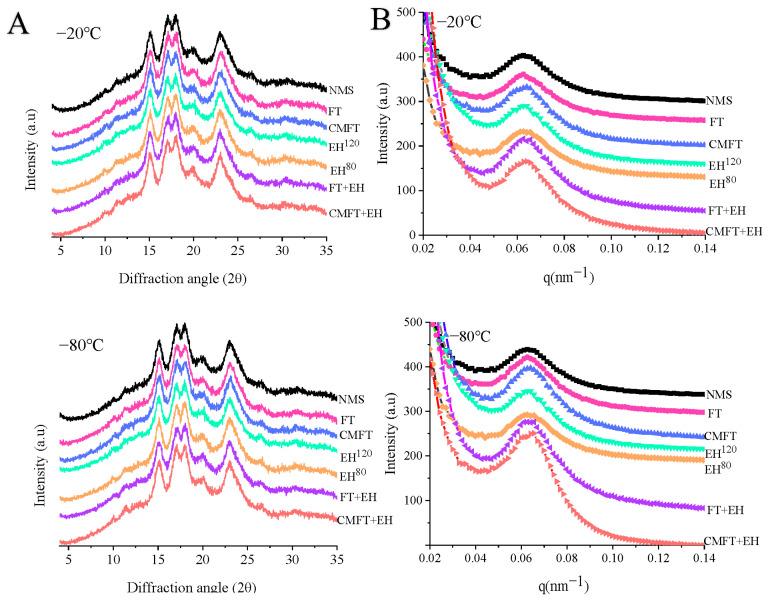

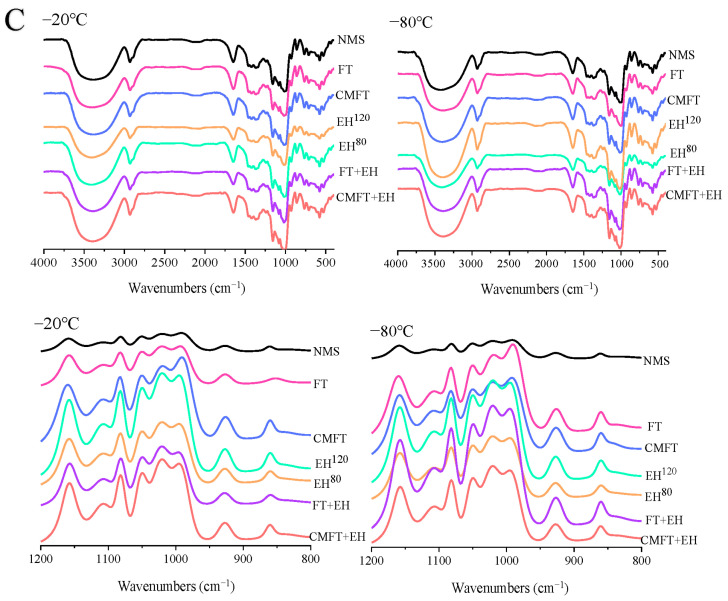
Long-range ordered (**A**), semi-layer structure (**B**), and short-range ordered structure (**C**) of porous starches. NMS indicates native maize starch. FT (freeze–thaw) and CMFT (critical melting with FT) indicate physical pretreatments before enzymatic hydrolysis. EH^120^ (control A: 120 U/g, 12 h, pH 6.0) and EH^80^ (control B: 80 U/g, 8 h, pH 6.0) indicate enzymatic hydrolysis alone. FT + EH and CMFT + EH indicate combined treatments. Freezing temperature includes −20 °C (slow freezing) and −80 °C (rapid freezing).

The XRD patterns and relative crystallinity (RC%) of native and porous starches are shown in Figure 3A and Table 5, respectively. Compared to NMS, all treatments significantly altered the diffraction peak intensity and RC%, with the most significant effects observed following combined treatments [8]. The RC% of NMS was 25.48%, whereas FT and CMFT pretreatments resulted in no significant changes or only a small increase in RC% (to ~26–27%). Enzymatic hydrolysis affected both amorphous and crystalline lamellae, thereby reducing overall crystallinity. Compared to NMS and FT-/CMFT-treated starches, EH^120^ decreased RC% to 24.74%, whereas EH^80^ increased RC% to 27.51% due to its limited extent of hydrolysis. Damage to starch crystalline regions is known to cause loss of structural integrity, thus affecting the adsorption properties of porous starch in practical applications [8,24]. Compared with NMS (25.48%) and enzymatic hydrolysis alone (~25%), the combined treatments of FT + EH and CMFT + EH significantly increased RC% to ~27.5% and 28.5%, respectively. Partial weakening of the inner structure by FT and CMFT pretreatments promoted effective hydrolysis in amorphous regions while facilitating reassociation of starch chains into ordered structures. Furthermore, applying FT or CMFT prior to enzymatic hydrolysis removed weaker amorphous regions, resulting in starch with a more perfected crystal structure [25]. These findings suggested that synergistic effects of the FT + EH and CMFT + EH treatments increased relative crystallinity while preserving A-type crystalline structure. The reassociation of starch chains, facilitated by these treatments, is critical for forming thermally stable networks within porous starch.

### 3.7. Lamellar Structure of Porous Starch

The semi-crystalline lamellar structures and fractal characteristics of native and porous starches are presented in Figure 3B and Table 5, respectively. The d-value indicates the lamellar thickness of starch. Meanwhile, an increased d-value indicated swelling of crystalline domains and weakening of hydrogen bonds caused by water/molecular intercalation between starch chains. This process expanded interlamellar spacing and enhanced molecular chain mobility, thereby facilitating the rearrangement of starch chains into new crystalline domains. As shown in Table 5, no significant differences occurred between NMS (9.92 nm), FT (9.93 nm), EH^120^ (9.91 nm), and EH^80^ (9.93 nm), whereas CMFT pretreatment slightly increased the d-value to ~10.08 nm. Compared with NMS and FT/CMFT treatment, both FT + EH and CMFT + EH further increased the d-value to approximately 10.11–10.28 nm. This suggested that combined physical-enzymatic strategies enhanced molecular chain mobility, facilitating rearrangement and aggregation into thicker crystalline domains.

Peak area (Ap) reflects the degree of structural order within starch granules [26]. Compared with NMS (Ap = 1.26), both FT and CMFT increased Ap to approximately 1.27–1.68. EH^80^ increased Ap to 1.75, while EH^120^ decreased it to 1.15 due to extensive structural damage. Compared with NMS and FT/CMFT treatment, the combined treatments of FT + EH and CMFT + EH further raised Ap to 1.9 and 2.91, respectively. These increases indicated that pretreatments promoted the formation of more ordered crystalline regions and were consistent with the increased RC% values shown in Table 5. Fractal analysis showed that NMS (α = 3.18) exhibited surface fractal characteristics (Ds). All treatments except EH^120^ and EH^80^ increased the α value, indicating denser and more ordered aggregate structures. The CMFT + EH treatment showed the highest α value (3.57) followed by FT + EH (3.44), indicating that combined treatments optimized structural hierarchy by promoting molecular rearrangement.

### 3.8. Short-Range Molecular Orders of Porous Starch

The FTIR spectra and the ratios of short-range molecular orders for native and porous starches are presented in Figure 3C and Table 5, respectively. No shifts in starch characteristic absorption peaks were observed after treatments, indicating that neither single nor combined treatments altered the inherent chemical structure or functional groups of starch. The ratio R (_1047/1022_ cm^−1^) reflects the degree of short-range order within starch granules and is positively correlated with structural regularity. Compared with NMS (1.088 cm^−1^), FT increased the R value to approximately 1.13, while CMFT further increased it to approximately 1.15. Slow freezing at −20 °C slightly decreased R to 1.057 (FT) and 1.079 (CMFT) primarily due to ice crystal-induced disruption of crystalline regions. In contrast, rapid freezing at −80 °C significantly increased the R value to 1.132 (FT) and 1.155 (CMFT), indicating enhanced ordering of starch chains within granules. These trends agreed well with the results shown in XRD and SAXS.

Compared with NMS and FT/CMFT treatments, EH^120^ decreased the R value to 1.037, whereas EH^80^ increased it to 1.161. The reduction for EH^120^ resulted from excessive hydrolysis disrupting starch short-range order [27]. Conversely, the increase for EH^80^ occurred because limited hydrolysis preferentially removed amorphous regions, increasing the relative order of residual crystalline structures. Combined treatments of FT + EH and CMFT + EH further increased the R value to 1.240 (−80 °C) and 1.307 (−80 °C), respectively. These enhancements were attributed to FT and CMFT pretreatments, which improved enzyme accessibility to amorphous regions for targeted hydrolysis and caused dissolved starch chains to release for structural reorganization, promoting stabilized crystalline domain formation [28,29].

### 3.9. Curcumin Encapsulation Efficiency (EE) and Encapsulation Capacity (EC)

**Figure 4:**
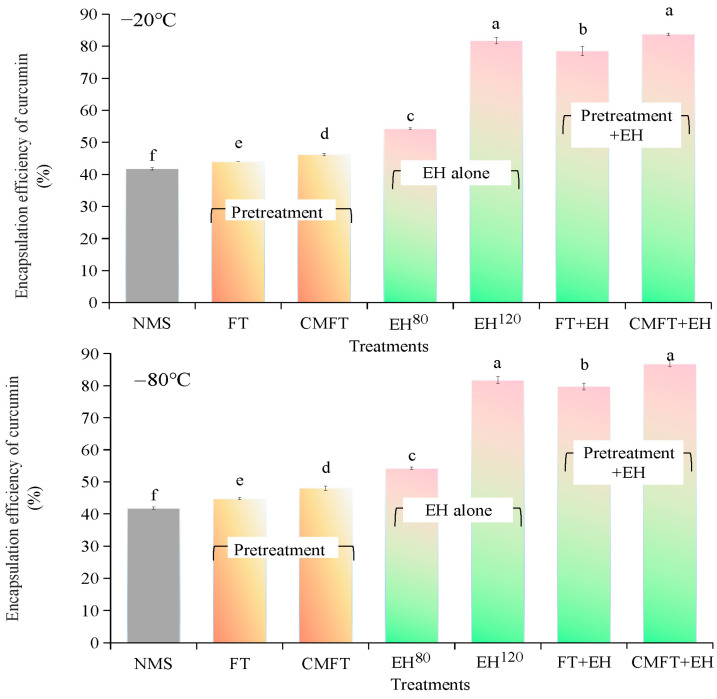

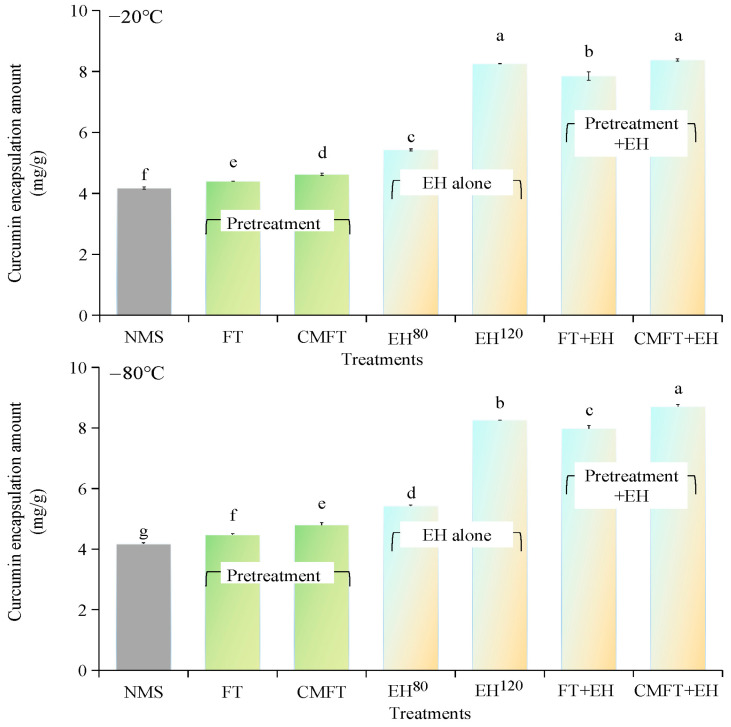
Encapsulation efficiency (EE, %) and encapsulation capacity (EC, mg/g) of porous starches. Different lowercase superscripts (a–g) within bars indicate significant differences among treatments. NMS indicates native maize starch. FT (freeze–thaw) and CMFT (critical melting with FT) indicate physical pretreatments before enzymatic hydrolysis. EH^120^ (control A: 120 U/g, 12 h, pH 6.0) and EH^80^ (control B: 80 U/g, 8 h, pH 6.0) indicate enzymatic hydrolysis alone. FT + EH and CMFT + EH indicate combined treatments. Freezing temperature includes −20 °C (slow freezing) and −80 °C (rapid freezing).

Figure 4 presents the encapsulation efficiency (EE) and encapsulation capacity (EC) of native and porous starches. The EE% and EC of NMS were 40% and 4.18 mg/g, respectively. All treatments increased curcumin encapsulation capabilities due to porous structures’ development within starch granules [30]. FT and CMFT slightly increased EE% and EC, whereas enzymatic hydrolysis alone significantly increased these values from 40% and 4.18 mg/g (NMS) to 81% and 7.8 mg/g (EH^120^) and 54% and 5.43 mg/g (EH^80^), respectively. Starch frozen at −80 °C improved encapsulation capacity more effectively than at −20 °C.

In contrast, the combined treatments further increased EE% and EC to 78% and 7.8 mg/g (FT + EH) and 88% and 8.70 mg/g (CMFT + EH). This enhancement was attributed to significantly increasing specific surface area and pore structures after FT + EH and CMFT + EH, facilitating curcumin loading within the porous network. Furthermore, the preserved granular morphology of porous starch further promoted curcumin encapsulation. Notably, EH^120^ increased EE% to 81% under the conditions of 120 U/g of enzyme addition for 12 h hydrolysis; however, FT + EH and CMFT + EH with only 80 U/g enzyme addition for 8 h hydrolysis increased EE% to 78% and 88%. Although this study did not evaluate pilot-scale processes, the reduced enzyme requirement and processing time suggested a potentially more cost-effective and scalable method for industrial applications.

### 3.10. Curcumin Stability Encapsulated in Porous Starch at Different Storage Times and Temperatures

Table 6 presents curcumin retention rates (%) for free and encapsulated forms as stored at 40–120 °C and for 5–20 d. Since WMS lacked porous structures, curcumin primarily adhered to granule surfaces, resulting in rapid release. FT and CMFT pretreatments showed slightly lower retention rates than NMS. In contrast, curcumin encapsulated within porous starch prepared by EH^120^ retained 56% after storage for 20 d and retained 78% after storage at 120 °C, whereas EH^80^ retained 60.79% and 79.77%, respectively. Formation of the porous structure in starch acted as a physical protective barrier for curcumin encapsulation. Compared with enzymatic hydrolysis alone, FT + EH and CMFT + EH further increased curcumin retention. When stored at 120 °C, CMFT + EH retained ~90% of curcumin, FT + EH retained 88%, and EH^120^ and EH^80^ retained ~78%, while the free form retained only 42.27%. This enhancement primarily resulted from the increased pore structures and preserved granular integrity, which collectively increased surface area and pore volume for curcumin encapsulation, while inhibiting starch dissolution during heating to preserve curcumin. Conversely, extensive granule damage in EH^120^ and limited pore development in EH^80^ reduced encapsulation capacity, promoting curcumin release.

These findings highlighted that combined treatments increased curcumin retention through two mechanisms: (1) pore structure formation enhancing curcumin encapsulation and (2) preserved granular integrity resisting thermal degradation. Furthermore, as discussed previously, FT and CMFT pretreatments thickened the starch matrix by partially weakening internal structures and modifying granule surfaces, promoting reassociation of dissolved starch chains into a compact and thermally stable matrix for degradation.

## 4. Conclusions

**Figure 5:**
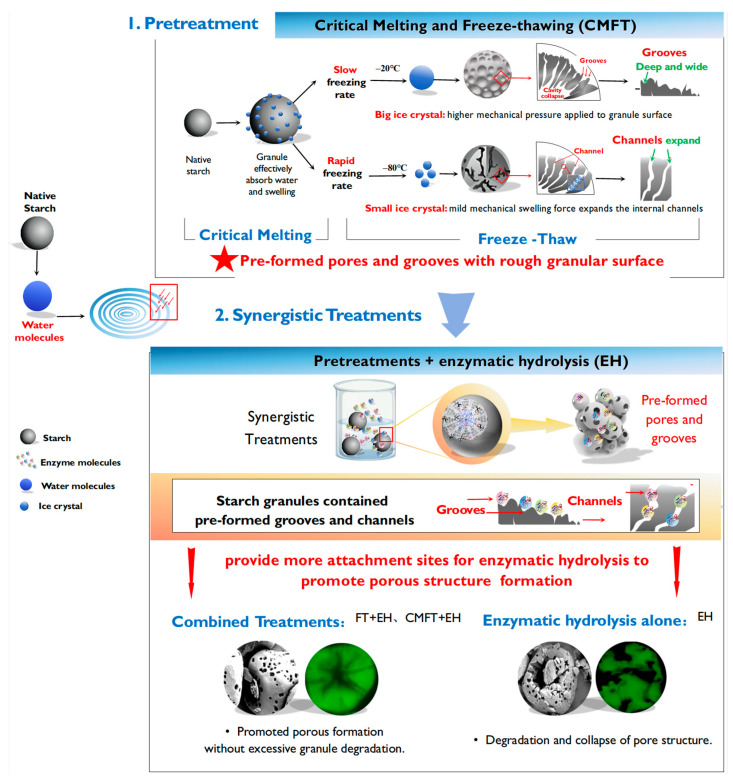
Mechanism of CMFT with enzymatic hydrolysis on porous starch preparation.

In this work, FT and CMFT prior to enzymatic hydrolysis can be used as an effective strategy to enhance enzymatic hydrolysis (EH) for porous starch preparation. FT and CMFT pretreatments increased enzyme attachment sites by weakening the starch inner structures and creating surface grooves and pores, thereby enhancing enzyme accessibility to facilitate porous structure formation while maintaining granular integrity (Figure 5). Freezing at −80 °C generated more extensive porous structures than freezing at −20 °C due to differing ice crystal formation mechanisms, especially for CMFT. The combined treatment of FT + EH, particularly CMFT + EH, significantly reduced enzyme requirements, with 33% lower enzyme addition and enzymatic hydrolysis time for porous starch preparation compared with EH (single enzymatic hydrolysis). The increased pore structures and preserved granular integrity by FT + EH and CMFT + EH increased curcumin encapsulation efficiency and curcumin retention under different storage conditions. This study provided a sustainable and efficient method for producing stable porous starch by combining CMFT pretreatment with enzymatic hydrolysis, addressing the limitations of conventional enzymatic methods. The combined treatment holds strong promise for industrial applications, especially in functional food delivery systems and bioactive encapsulation, owing to its scalability, cost-effectiveness, and stability-enhancing effects.

## Figures and Tables

**Table 1:** Experimental design and corresponding nomenclature.

Experimental Design	Nomenclature	Hydrolysis Conditions
Native normal maize starch	NMS	-
Physical pretreatments	FT and CMFT	-
Native starch enzymatically hydrolyzed at its optimal hydrolysis conditions	EH^120^ (Control A)	120 U/g, 12 h, pH 6.0
Native starch enzymatically hydrolyzed at the optimal conditions based on FT- and CMFT-pretreated starches	EH^80^ (Control B)	80 U/g, 8 h, pH 6.0
FT- and CMFT-pretreated starch hydrolyzed at its optimal hydrolysis conditions	FT + EH and CMFT + EH	80 U/g, 8 h, pH 6.0

**Table 2:** Specific surface area, pore volume, and average pore diameter of starch.

Treatments		Freezing Temperature (°C)	Specific Surface Area (m^2^/g)	Volume of Mesopores (cm^3^/g) × 10^−3^	Average Diameter of Mesopores (nm)
-	NMS	-	1.01 ± 0.01 ^e^	1.60 ± 0.49 ^d^	2.99 ± 0.02 ^a^
Pretreatment	FT	−20 °C	1.10 ± 0.12 ^e^	2.53 ± 0.39 ^d^	3.21 ± 0.00 ^a^
		−80 °C	1.71 ± 0.05 ^e^	3.98 ± 1.91 ^d^	3.06 ± 0.12 ^a^
	CMFT	−20 °C	3.47 ± 0.03 ^d^	3.77 ± 0.07 ^d^	3.24 ± 0.38 ^a^
		−80 °C	4.19 ± 0.24 ^d^	4.14 ± 1.09 ^d^	3.37 ± 0.05 ^a^
EH alone	EH^120^	-	17.00 ± 0.92 ^b^	15.97 ± 2.55 ^b^	3.44 ± 0.43 ^a^
	EH^80^	-	6.63 ± 0.95 ^c^	7.34 ± 1.48 ^c^	3.23 ± 0.13 ^a^
Pretreatment + EH	FT + EH	−20 °C	7.00 ± 1.22 ^c^	8.63 ± 0.14 ^c^	3.34 ± 0.14 ^a^
		−80 °C	8.89 ± 1.45 ^c^	8.38 ± 0.48 ^d^	3.47 ± 0.05 ^a^
	CMFT + EH	−20 °C	15.13 ± 1.43 ^b^	16.53 ± 0.27 ^b^	3.52 ± 0.02 ^a^
		−80 °C	26.76 ± 1.86 ^a^	21.66 ± 2.86 ^a^	3.64 ± 0.14 ^a^

All data represent mean ± SD (n ≥ 3). Different lowercase superscripts (a–e) within the same column indicate significant differences among treatments. NMS indicates native maize starch. FT (freeze–thaw) and CMFT (critical melting with FT) indicate physical pretreatments before enzymatic hydrolysis. EH^120^ (control A: 120 U/g, 12 h, pH 6.0) and EH^80^ (control B: 80 U/g, 8 h, pH 6.0) indicate enzymatic hydrolysis alone. FT + EH and CMFT + EH indicate combined treatments. The freezing temperature includes −20 °C (slow freezing) and −80 °C (rapid freezing).

**Table 3:** Oil and water absorption of porous starches.

Treatments		Freezing Temperature (°C)	WA (%)	OA (%)
-	NMS	-	84.99 ± 1.80 ^f^	75.55 ± 2.93 ^d^
Pretreatment	FT	−20 °C	116.68 ± 1.58 ^e^	68.80 ± 1.78 ^e^
		−80 °C	120.93 ± 1.32 ^e^	68.29 ± 3.46 ^e^
	CMFT	−20 °C	136.76 ± 5.18 ^d^	74.19 ± 4.68 ^de^
		−80 °C	138.47 ± 1.74 ^d^	79.96 ± 3.53 ^d^
EH alone	EH^120^	-	154.25 ± 5.59 ^bc^	120.40 ± 0.06 ^b^
	EH^80^	-	121.05 ± 5.93 ^e^	94.38 ± 1.70 ^c^
Pretreatment + EH	FT + EH	−20 °C	153.85 ± 4.85 ^bc^	117.02 ± 3.93 ^b^
		−80 °C	148.11 ± 3.49 ^c^	120.79 ± 3.95 ^b^
	CMFT + EH	−20 °C	162.76 ± 7.52 ^ab^	130.23 ± 1.09 ^a^
		−80 °C	168.55 ± 0.44 ^a^	134.40 ± 0.31 ^a^

All data represent mean ± SD (n ≥ 3). Different lowercase superscripts (a–f) within the same column indicate significant differences among treatments. NMS indicates native maize starch. FT (freeze–thaw) and CMFT (critical melting with FT) indicate physical pretreatments before enzymatic hydrolysis. Different lowercase superscripts EH^120^ (control A: 120 U/g, 12 h, pH 6.0) and EH^80^ (control B: 80 U/g, 8 h, pH 6.0) indicate enzymatic hydrolysis alone. FT + EH and CMFT + EH indicate combined treatments. Freezing temperature includes −20 °C (slow freezing) and −80 °C (rapid freezing).

**Table 4:** Thermal properties of porous starch.

Treatments		Freezing Temperature (°C)	Gelatinization Temperature (°C)			Enthalpy (J/g)
			To	Tp	Tc	
-	NMS	-	63.74 ± 0.11 ^f^	67.95 ± 0.24 ^e^	72.47 ± 0.35 ^d^	10.81 ± 0.72 ^b^
Pretreatment	FT	−20 °C	64.24 ± 0.82 ^ef^	67.95 ± 0.24 ^e^	72.47 ± 0.35 ^d^	14.31 ± 0.01 ^a^
		−80 °C	65.13 ± 0.11 ^e^	69.39 ± 0.70 ^cde^	72.73 ± 0.89 ^cd^	13.10 ± 0.66 ^a^
	CMFT	−20 °C	67.99 ± 0.13 ^bc^	70.49 ± 0.35 ^bcd^	73.81 ± 0.68 ^bcd^	10.90 ± 0.19 ^b^
		−80 °C	68.57 ± 0.07 ^b^	71.78 ± 0.74 ^b^	74.46 ± 0.58 ^bc^	9.83 ± 0.19 ^c^
EH alone	EH^120^	-	66.78 ± 0.15 ^d^	70.73 ± 0.02 ^bc^	75.43 ± 1.03 ^b^	9.50 ± 0.40 ^c^
	EH^80^	-	65.17 ± 0.38 ^e^	69.08 ± 0.22 ^de^	73.06 ± 0.30 ^cd^	9.70 ± 1.40 ^c^
Pretreatment + EH	FT + EH	−20 °C	67.21 ± 1.00 ^cd^	71.92 ± 1.82 ^b^	75.29 ± 1.30 ^b^	10.83 ± 0.88 ^b^
		−80 °C	67.35 ± 0.06 ^cd^	69.95 ± 0.35 ^cd^	73.90 ± 1.10 ^bcd^	10.68 ± 0.89 ^b^
	CMFT + EH	−20 °C	69.71 ± 0.31 ^a^	73.69 ± 0.15 ^a^	79.04 ± 0.47 ^a^	10.36 ± 0.01 ^b^
		−80 °C	70.22 ± 0.20 ^a^	73.76 ± 0.08 ^a^	77.80 ± 0.97 ^a^	10.97 ± 1.10 ^b^

All data represent mean ± SD (n ≥ 3). Different lowercase superscripts (a–f) within the same column indicate significant differences among treatments. NMS indicates native maize starch. FT (freeze–thaw) and CMFT (critical melting with FT) indicate physical pretreatments before enzymatic hydrolysis. EH^120^ (control A: 120 U/g, 12 h, pH 6.0) and EH^80^ (control B: 80 U/g, 8 h, pH 6.0) indicate enzymatic hydrolysis alone. FT + EH and CMFT + EH indicate combined treatments. Freezing temperature includes −20 °C (slow freezing) and −80 °C (rapid freezing).

**Table 5:** Long-range ordered structure, short-range ordered structure, and semi-layer structure of porous starches.

Treatments		Freezing Temperature (°C)	RC (%)	R_1047/1022_	Lamellar Parameter				Fractal Features	
					q (nm^−1^)	d_Bragg_ (nm)	Imax (a.u.)	Ap	α	Ds
-	NMS	-	25.48 ± 0.08 ^e^	1.088 ± 0.023 ^ef^	0.63 ± 0.00 ^a^	9.92 ± 0.01 ^c^	112.42 ± 2.98 ^g^	1.26 ± 0.01 ^cde^	3.18 ± 0.01 ^e^	2.82 ± 0.01 ^c^
Pretreatment	FT	−20 °C	25.09 ± 0.05 ^ef^	1.057 ± 0.060 ^f^	0.63 ± 0.00 ^a^	9.93 ± 0.01 ^c^	104.23 ± 1.89 ^h^	1.06 ± 0.05 ^e^	3.22 ± 0.01 ^de^	2.78 ± 0.01 ^cd^
		−80 °C	26.65 ± 0.70 ^d^	1.132 ± 0.000 ^de^	0.63 ± 0.00 ^a^	9.93 ± 0.03 ^c^	132.38 ± 0.67 ^f^	1.54 ± 0.03 ^bcde^	3.36 ± 0.01 ^c^	2.64 ± 0.01 ^e^
	CMFT	−20 °C	25.44 ± 0.04 ^e^	1.079 ± 0.027 ^ef^	0.62 ± 0.00 ^b^	10.08 ± 0.01 ^b^	113.54 ± 1.61 ^g^	1.27 ± 0.01 ^cde^	3.25 ± 0.01 ^d^	2.75 ± 0.01 ^d^
		−80 °C	27.40 ± 0.01 ^c^	1.155 ± 0.007 ^d^	0.62 ± 0.00 ^b^	10.09 ± 0.03 ^b^	143.79 ± 2.67 ^e^	1.68 ± 0.02 ^bcd^	3.44 ± 0.04 ^b^	2.57 ± 0.04 ^f^
EH alone	EH^120^	-	24.74 ± 0.22 ^f^	1.037 ± 0.005 ^f^	0.63 ± 0.00 ^a^	9.91 ± 0.00 ^c^	112.98 ± 0.62 ^g^	1.15 ± 0.02 ^de^	3.01 ± 0.01 ^g^	2.99 ± 0.01 ^a^
	EH^80^	-	27.51 ± 0.02 ^c^	1.161 ± 0.003 ^d^	0.63 ± 0.00 ^a^	9.93 ± 0.01 ^c^	142.53 ± 2.84 ^e^	1.75 ± 0.04 ^bc^	3.13 ± 0.04 ^f^	2.88 ± 0.04 ^b^
Pretreatment + EH	FT + EH	−20 °C	27.50 ± 0.08 ^c^	1.177 ± 0.010 ^cd^	0.62 ± 0.00 ^b^	10.11 ± 0.01 ^b^	174.57 ± 0.95 ^d^	1.84 ± 0.01 ^b^	3.24 ± 0.01 ^de^	2.77 ± 0.01 ^cd^
		−80 °C	28.65 ± 0.03 ^b^	1.240 ± 0.023 ^b^	0.61 ± 0.00 ^c^	10.24 ± 0.01 ^a^	236.38 ± 0.69 ^b^	1.99 ± 0.72 ^b^	3.44 ± 0.04 ^b^	2.57 ± 0.04 ^f^
	CMFT + EH	−20 °C	28.59 ± 0.01 ^b^	1.216 ± 0.011 ^bc^	0.62 ± 0.00 ^b^	10.12 ± 0.02 ^b^	216.62 ± 1.80 ^c^	2.50 ± 0.11 ^a^	3.35 ± 0.04 ^c^	2.65 ± 0.04 ^e^
		−80 °C	29.75 ± 0.33 ^a^	1.307 ± 0.019 ^a^	0.61 ± 0.00 ^c^	10.28 ± 0.01 ^a^	261.93 ± 0.37 ^a^	2.91 ± 0.04 ^a^	3.57 ± 0.01 ^a^	2.44 ± 0.01 ^g^

All data represent mean ± SD (n ≥ 3). Different lowercase superscripts (a–h) within the same column indicate significant differences among treatments. NMS indicates native maize starch. FT (freeze–thaw) and CMFT (critical melting with FT) indicate physical pretreatments before enzymatic hydrolysis. EH^120^ (control A: 120 U/g, 12 h, pH 6.0) and EH^80^ (control B: 80 U/g, 8 h, pH 6.0) indicate enzymatic hydrolysis alone. FT + EH and CMFT + EH indicate combined treatments. Freezing temperature includes −20 °C (slow freezing) and −80 °C (rapid freezing).

**Table 6:** Curcumin stability encapsulated in porous starch at different storage times and temperatures.

Treatments		Freezing Temperature (°C)	Curcumin Retention Rate (%)				Curcumin Retention Rate (%)			
			5 d	10 d	15 d	20 d	40 °C	60 °C	80 °C	120 °C
-	Cur	-	80.12 ± 1.59 ^g^	69.56 ± 0.93 ^h^	57.68 ± 3.46 ^c^	47.65 ± 1.07 ^d^	88.17 ± 1.35 ^b^	74.39 ± 1.20 ^c^	60.55 ± 2.34 ^d^	42.27 ± 1.16 ^e^
-	NMS-Cur		83.23 ± 1.22 ^fg^	72.23 ± 0.20 ^gh^	61.73 ± 0.51 ^c^	50.95 ± 0.74 ^cd^	95.67 ± 0.56 ^a^	90.26 ± 0.20 ^b^	85.58 ± 0.41 ^bc^	70.87 ± 0.78 ^d^
Pretreatment	FT-Cur	−20 °C	85.83 ± 1.18 ^ef^	75.08 ± 2.47 ^fg^	67.58 ± 3.42 ^b^	51.58 ± 2.47 ^cd^	95.83 ± 1.18 ^a^	92.50 ± 1.18 ^ab^	87.50 ± 1.18 ^bc^	78.33 ± 0.00 ^c^
		−80 °C	86.61 ± 1.26 ^def^	76.79 ± 2.53 ^f^	70.71 ± 1.01 ^b^	54.20 ± 4.17 ^c^	96.16 ± 3.41 ^a^	92.86 ± 4.04 ^ab^	88.04 ± 0.76 ^b^	79.64 ± 0.18 ^bc^
	CMFT-Cur	−20 °C	87.50 ± 3.54 ^cdef^	76.73 ± 1.80 ^f^	69.95 ± 1.35 ^b^	56.77 ± 3.15 ^bc^	97.23 ± 2.51 ^a^	93.91 ± 2.96 ^ab^	88.45 ± 3.47 ^b^	81.91 ± 0.13 ^bc^
		−80 °C	89.01 ± 4.66 ^cde^	77.75 ± 1.17 ^ef^	70.33 ± 1.55 ^b^	56.57 ± 3.85 ^bc^	97.47 ± 2.49 ^a^	94.45 ± 0.23 ^ab^	88.80 ± 3.73 ^b^	82.94 ± 0.90 ^b^
EH alone	EH^120^-Cur	-	89.62 ± 3.81 ^bcde^	80.38 ± 0.54 ^de^	68.62 ± 0.87 ^b^	56.92 ± 4.35 ^bc^	97.42 ± 2.01 ^a^	94.08 ± 2.94 ^ab^	82.88 ± 2.82 ^c^	78.46 ± 2.18 ^c^
	EH^80^-Cur	-	89.09 ± 0.34 ^cde^	82.36 ± 0.44 ^cd^	71.95 ± 3.84 ^b^	60.79 ± 0.76 ^b^	97.36 ± 1.92 ^a^	94.74 ± 1.99 ^ab^	87.16 ± 1.83 ^bc^	79.77 ± 2.22 ^bc^
Pretreatment + EH	FT + EH −Cur	−20 °C	92.20 ± 2.72 ^abcd^	85.01 ± 0.40 ^bc^	78.55 ± 0.87 ^a^	71.41 ± 1.16 ^a^	97.88 ± 0.92 ^a^	94.98 ± 1.21 ^ab^	93.59 ± 0.75 ^a^	87.91 ± 2.54 ^a^
		−80 °C	93.10 ± 0.20 ^abc^	86.23 ± 1.55 ^b^	79.95 ± 3.51 ^a^	72.46 ± 3.10 ^a^	98.62 ± 0.04 ^a^	95.21 ± 2.79 ^ab^	94.51 ± 1.80 ^a^	88.29 ± 0.63 ^a^
	CMFT + EH −Cur	−20 °C	95.04 ± 1.71 ^ab^	88.19 ± 0.98 ^ab^	82.61 ± 0.15 ^a^	75.15 ± 0.22 ^a^	98.14 ± 0.86 ^a^	96.89 ± 0.91 ^a^	95.03 ± 0.04 ^a^	89.45 ± 2.54 ^a^
		−80 °C	95.92 ± 0.89 ^a^	89.55 ± 1.47 ^a^	83.75 ± 3.02 ^a^	77.34 ± 2.09 ^a^	98.25 ± 0.85 ^a^	97.09 ± 0.87 ^a^	95.35 ± 0.08 ^a^	90.68 ± 1.80 ^a^

All data represent mean ± SD (n ≥ 3). Different lowercase superscripts (a–h) within the same column indicate significant differences among treatments. NMS indicates native maize starch. FT (freeze–thaw) and CMFT (critical melting with FT) indicate physical pretreatments before enzymatic hydrolysis EH^120^ (control A: 120 U/g, 12 h, pH 6.0), and EH^80^ (control B: 80 U/g, 8 h, pH 6.0) indicates enzymatic hydrolysis alone. FT + EH and CMFT + EH indicate combined treatments. Freezing temperature includes −20 °C (slow freezing) and −80 °C (rapid freezing).

## Data Availability

The original contributions presented in this study are included in the article. Further inquiries can be directed to the corresponding author.

## Supplementary Materials

The following supporting information can be downloaded at: https://www.mdpi.com/article/10.3390/foods14172984/s1, Figure S1. Effect of enzyme addition on the yield, water and oil absorption of normal maize starch (A) and pore-forming properties (B) of starch; Figure S2. Effect of enzyme time on the yield, water and oil absorption of normal maize starch (A) and pore-forming properties (B) of starch; Figure S3. Effect of pH on the yield, water and oil absorption of normal maize starch (A) and pore-forming properties (B) of starch.
