# Supplementary material for: Critical Melting–Freezing Pretreatment Enhances Enzymatic Hydrolysis for Porous Starch Preparation: Role of Partial Structural Weakening and Surface Modification
Source: Foods. 2025 Aug 26;14(17):2984. doi: 10.3390/foods14172984 (PMC12428241; doi:10.3390/foods14172984)
Supplement: Supplementary file 1 [file foods-14-02984-s001.zip › foods-3807158-supplementary.pdf]

Appendix I

A

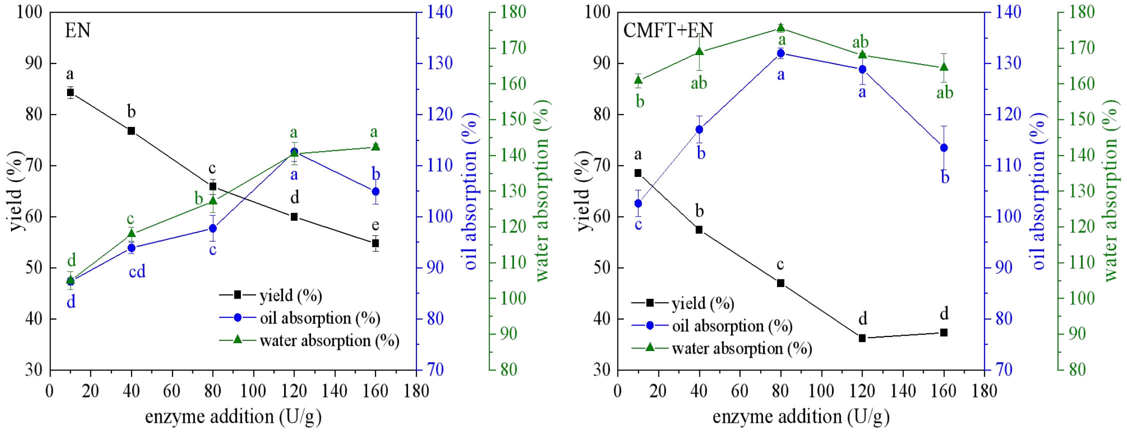

B

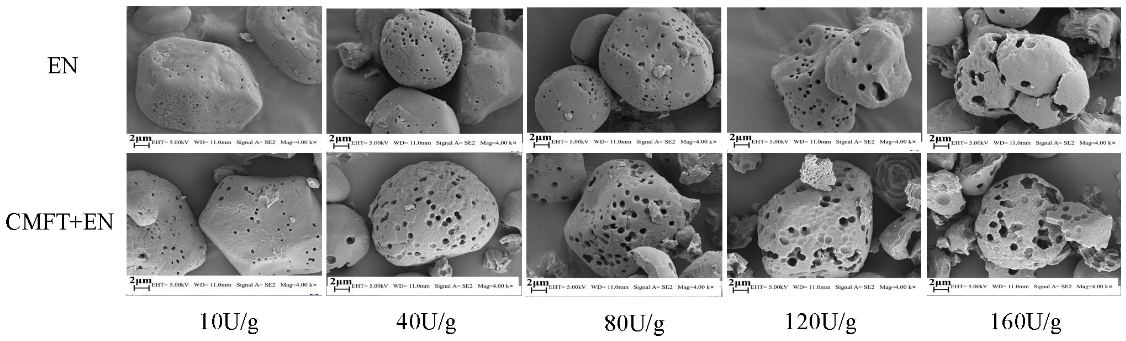

Supplementary Fig.I Effect of enzyme addition on the yield, water and oil absorption of normal maize starch (A) and pore-forming properties (B) of starch

Appendix II

A

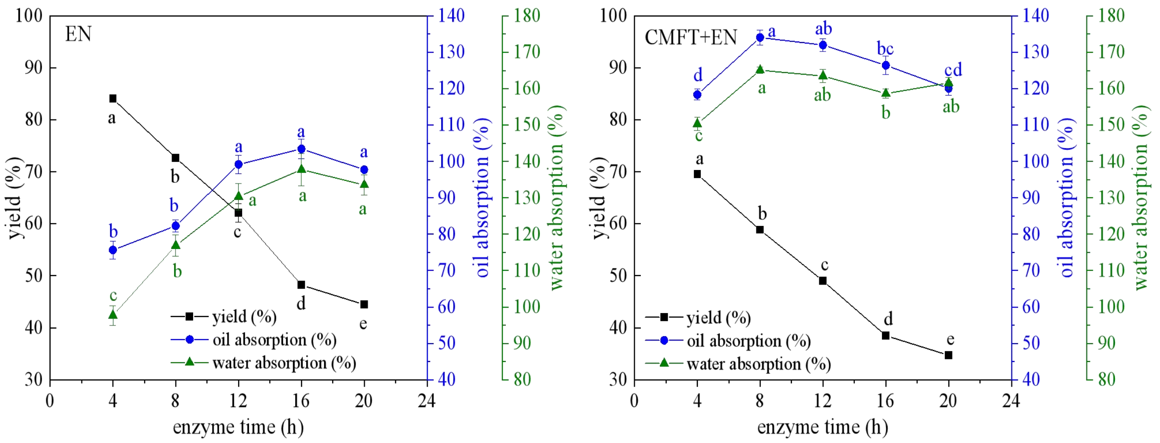

B

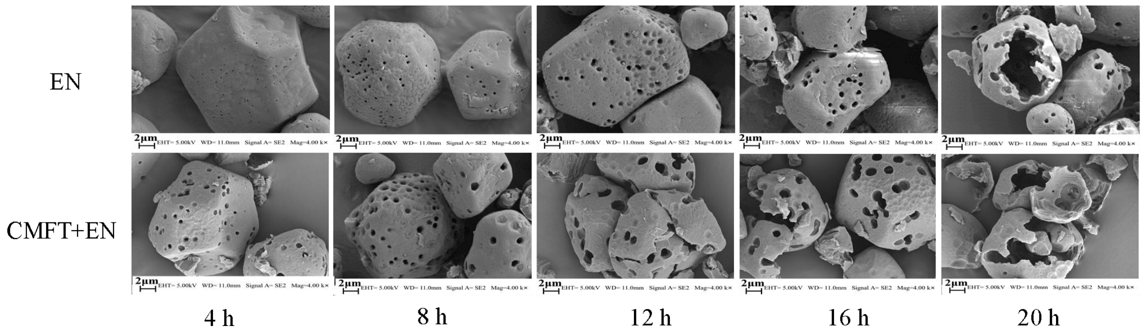

Supplementary Fig. II Effect of enzyme time on the yield, water and oil absorption of normal maize starch (A) and pore-forming properties (B) of starch

Appendix III

A

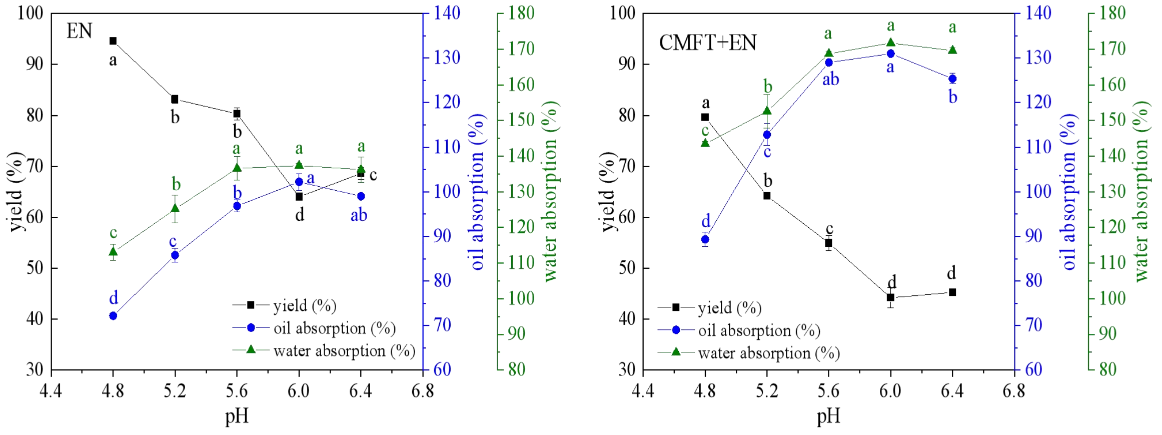

B

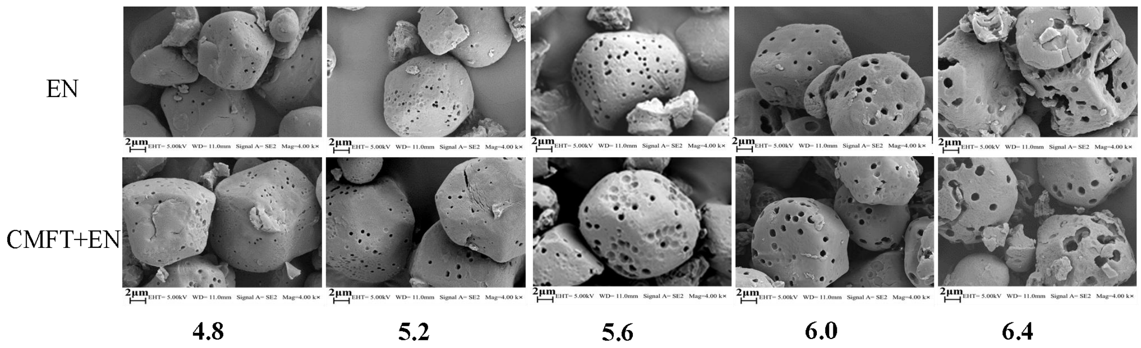

Supplementary Fig.III Effect of pH on the yield, water and oil absorption of normal maize starch (A) and pore-forming properties (B) of starch
